# Effects of Low Doses of L-Carnitine Tartrate and Lipid Multi-Particulate Formulated Creatine Monohydrate on Muscle Protein Synthesis in Myoblasts and Bioavailability in Humans and Rodents

**DOI:** 10.3390/nu13113985

**Published:** 2021-11-09

**Authors:** Roger A. Fielding, Donato Rivas, Gregory J. Grosicki, Yassine Ezzyat, Lisa Ceglia, Lori Lyn Price, Cemal Orhan, Kazim Sahin, Kelli Fowler, Tyler White, Shane Durkee, Katja Kritsch, Aouatef Bellamine

**Affiliations:** 1 Nutrition, Exercise Physiology, and Sarcopenia Laboratory, Jean Mayer USDA Human Nutrition Research Center, Aging Tufts University, Boston, MA 02111, USA; donato.rivas@tufts.edu (D.R.); ggrosicki@georgiasouthern.edu (G.J.G.); yassine.ezzyat@tufts.edu (Y.E.); 2 Biodynamics and Human Performance Center, Georgia Southern University, Armsrong Campus, Savannah, GA 31419, USA; 3 Division of Endocrinology, Tufts Medical Center, Boston, MA 02111, USA; lceglia@tuftsmedicalcenter.org; 4 The Institute for Clinical Research and Health Policy Studies, Tufts Medical Center, Boston, MA 02111, USA; lprice1@tuftsmedicalcenter.org; 5 Tufts Clinical and Translational Science Institute, Tufts University, Boston, MA 02111, USA; 6 Department of Animal Nutrition, Faculty of Veterinary Medicine, Firat University, Elazig 23119, Turkey; corhan@firat.edu.tr (C.O.); nsahinkm@yahoo.com (K.S.); 7 R&D Innovation, Lonza Consumer Health, Morristown, NJ 07960, USA; kelli.fowler@lonza.com (K.F.); Tyle.white@lonza.com (T.W.); shane.durkee@lonza.com (S.D.); aouatef.bellamine@lonza.com (A.B.); 8 R&D Lonza Specialty Ingredients, Alpharetta, GA 30004, USA; katja.kritsch@lonza.com

**Keywords:** muscle, carnitine, creatine, protein synthesis

## Abstract

The primary objective of this study was to investigate the potential synergy between low doses of L-carnitine tartrate and creatine monohydrate to induce muscle protein synthesis and anabolic pathway activation in primary human myoblasts. In addition, the effects of Lipid multi-particulates (LMP) formulation on creatine stability and bioavailability were assessed in rodents and healthy human subjects. When used individually, L-carnitine tartrate at 50 µM and creatine monohydrate at 0.5 µM did not affect myoblast protein synthesis and signaling. However, when combined, they led to a significant increase in protein synthesis. Increased AKT and RPS6 phosphorylation were observed with 50 µM L-carnitine tartrate 5 µM creatine in combination in primary human myoblasts. When Wistar rats were administered creatine with LMP formulation at either 21 or 51 mg/kg, bioavailability was increased by 27% based on the increase in the area under the curve (AUC) at a 51 mg/kg dose compared to without LMP formulation. Tmax and Cmax were unchanged. Finally, in human subjects, a combination of LMP formulated L-carnitine at 500 mg (from L-carnitine tartrate) with LMP formulated creatine at 100, 200, or 500 mg revealed a significant and dose-dependent increase in plasma creatine concentrations. Serum total L-carnitine levels rose in a similar manner in the three combinations. These results suggest that a combination of low doses of L-carnitine tartrate and creatine monohydrate may lead to a significant and synergistic enhancement of muscle protein synthesis and activation of anabolic signaling. In addition, the LMP formulation of creatine improved its bioavailability. L-carnitine at 500 mg and LMP-formulated creatine at 200 or 500 mg may be useful for future clinical trials to evaluate the effects on muscle protein synthesis.

## 1. Introduction

Both exercise and age influence skeletal muscle protein turnover. Protein accretion is the result of protein degradation and de novo protein synthesis [1]. During physical activity, biochemical processes such as hypoxia of the muscle, tissue degradation, free radical formation, and sarcolemma disruption may occur and can be prevented by nutritional supplements [2]. With age, the decrease in skeletal muscle size and loss of function leads to clinically relevant complaints, including progressive strength loss, fatigue, and lack of mobility [3]. Significant progress has been made in the comprehension of the molecular mechanisms underlying muscle atrophy. However, the prevention of muscle dysfunction with established treatment protocols is primarily symptom-oriented and includes physical therapy and exercise, but no specifically approved pharmacologic interventions are currently available [4,5]. Considering the lack of therapies for age-related muscle atrophy, the idea that nutritional supplements might have beneficial effects on muscle growth is of renewed interest [6].

L-carnitine and creatine are used as nutritional supplements and have been shown to positively affect muscle protein metabolism, typically at relatively high doses (L-carnitine 1–3 g/day; creatine 5–20 g/day) [7]. L-carnitine is a quaternary amine that plays an essential role in cellular energy metabolism due to the acylation of its β-hydroxyl group and its role in the long- and medium-chain fatty acid transport into the mitochondrial matrix, where the latter undergo β-oxidation, resulting in energy production and adenosine triphosphate (ATP) formation. Creatine helps to supply energy to all cells in the body, primarily muscle, thus biochemically providing the energy supply to phosphorylate adenosine diphosphate (ADP) to ATP during and following intense energy demand. This supply is largely dependent on the amount of phosphocreatine (PCr) stored in the muscle [8]. As PCr stores become depleted, energy availability diminishes due to the inability to resynthesize ATP at the rate required to sustain energy demand [8]. Because of its low stability at low pH during its gastrointestinal transit [9,10,11], creatine is usually ingested in high doses, 20 g dose for a week and 5 g dose daily subsequently. However, creatine biosynthesis and metabolism are highly dependent on many factors such as diet, hormones, and age [11,12]. In addition, it has been reported that high-dose creatine supplementation may reduce its absorption by down-regulating its transporter [13].

The aim of the present study was to examine whether L-carnitine tartrate and creatine monohydrate with or without LMP formulation specifically induce optimal protein synthesis. We specifically wanted to examine whether L-carnitine and creatine combined and at low doses can induce a synergistic and significant increase in skeletal muscle protein synthesis and activate anabolic signaling pathways in human primary myoblasts. We sought to determine the lowest dose of L-carnitine and creatine to elicit these promyogenic effects. We hypothesized that L-carnitine, by providing a constant production of ATP molecules, can stimulate PCr formation, leading to a low optimal efficacious dose of creatine [14]. To translate these findings, we conducted a bioavailability experiment in rodents to evaluate the effect of a lipid multi-particulate (LMP) technology on protecting the pH-related degradation and enhancing the serum bioavailability of creatine. We then examined the pharmacokinetics of low variable doses of LMP-formulated L-carnitine and creatine in healthy human subjects.

## 2. Materials and Methods

### 2.1. Materials

Carnipure tartrate (CARN) consisting of 68% elemental carnitine and 32% L-tartaric acid was provided by Lonza Consumer Health Inc. Creatine monohydrate (Cr) was purchased from Shanghai Baosui Chemical Co., Ltd. (Shanghai, China) LMP L-carnitine tartrate, and creatine were formulated at the Lonza site in Greenwood, SC, United States. Briefly, the compounds were combined with candelilla wax (14%), Stearyl Alcohol (17%), Stearic Acid (16%), and Sunflower Lecithin (2%). This mixture was melted and blended at 65–70 °C and sprayed with the melt-spray congeal technology to form the lipid multi-particulates. Recombinant human insulin-like growth factor-1 (IGF-1) and Dulbecco’s Modified Eagle Medium/Nutrient Mixture F-12 (DMEM-F12), Tryp-LE, Horse Serum, and phosphate-buffered saline (PBS) were purchased from Life Technologies (Thermo Fisher Scientific, Waltham, MA, USA). Puromycin and α-puromycin Ab (Cat# MABE343) were purchased from Sigma-Aldrich (St. Louis, MO, USA). Phospho-protein kinase B (phospho-AKT) (Cat #9271), phospho-ribosomal protein S6 (phopho-RPS6K1) (Cat# #2215), and Glycerinaldehyde-3-phosphate-Dehydrogenase (GAPDH) (Cat #4691) were purchased from Cell Signaling Technologies (Danvers, MA, USA). For the liquid chromatography with tandem mass spectrometry (LC/MS-MS) analysis, L-carnitine inner salt, creatine monohydrate, L-carnitine-(trimethyl-d9)-inner salt, and creatine-(methyl-d3) monohydrate were purchased from Sigma-Aldrich (St. Louis, MO, USA).

### 2.2. Muscle Cell Culture Experiments

Human skeletal muscle myoblast (HSMM) progenitors derived from 6 healthy males were obtained from Lonza and maintained at 37 °C (95% O_2_-5% CO_2_) in SkGMTM-2 Skelet al. Muscle Cell Growth Medium (Cat# CC-3245, Lonza). HSMMs are isolated from normal donors. HSMMs are isolated from the upper arm or leg muscle tissue and are sold in second passage. For experimental procedures, the myoblasts were seeded in 12-well plates and treated with several doses of Cr or CARN individually or in combination. After 22 h of incubation with Cr/CARN, cells were subjected to a 1.5 h serum-free starve, after which the media and appropriate stimulus were reapplied along with puromycin (1 μM). After 30 min of puromycin treatment, cells were lysed in RIPA Lysis and Extraction Buffer (Cat# 89901) that included Halt Protease Inhibitor Cocktail (Cat# 78430) and Halt Phosphatase Inhibitor Cocktail (Cat# 78420) from Thermo Fisher Scientific (Waltham, MA, USA). Each dose was performed in triplicate for each of the six subjects.

### 2.3. Protein Synthesis and Phosphorylation

Protein synthesis and anabolic signaling measures were made at the 24 h time point for all conditions. Protein synthesis was determined by measuring puromycin incorporation using SUnSET technique [15]. Anabolic signaling was determined as the quantity of pAKT and pRPS6 normalized to Glycerinaldehyde-3-phosphate-Dehydrogenase (GAPDH). For total protein determination, tissue lysates were solubilized in Laemmli buffer (BioRad, Hercules, CA, USA) and separated by SDS-PAGE using precast Tris·HCl gels (BioRad, Hercules, CA, USA). Protein was transferred to polyvinylidene fluoride membranes (BioRad, Hercules, CA, USA).

### 2.4. Bioavailability of LMP-Formulation Creatine in Rodents

Male Wistar rats (age = 8 weeks) (mean weight of 180 ± 20 g) were purchased from Firat University Experimental Research Center (Elazig, Turkey). Animals were kept in an isolated room at a constant temperature between 21 and 23 °C, controlled humidity (50 + 10%), and subjected to cycles of 12 h: artificial light/darkness. The study was approved by the Animal Ethics Committee of Firat University (27/05/2020-393056) and conducted in accordance with the standard ethical guidelines for laboratory animal use and care as defined in the European Economic Community rules. Rats (*n* = 7 per group) were randomly assigned into five groups: control and a group for each of the creatine and LMP creatine doses. Rats were fasted for 12 h with free access to water and orally administered by gastric intubation (5 mL/kg BW) as follows: (1) Control group: water; (2) Creatine (CRE) group 1 (CRE21): 30% *w*/*v* solution of 21 mg/kg BW of creatine; (3) CRE group 2 (CRE51): 30% *w*/*v* solution of 51 mg/kg BW of creatine; (4) LMP creatine group 1 (LMP CRE21): 30% *w*/*v* solution of 21 mg/kg BW of LMP creatine; (5) LMP creatine group 2 (LMP CRE51): 30% *w*/*v* solution of 51 mg/kg BW of LMP creatine. Blood was collected 15 min before oral gavage and after the corresponding oral administration of each group at different time-lapses (30, 60, 90, 120, and 180 min). Blood levels of creatine were measured using high-performance liquid chromatography (HPLC) (Shimadzu Co Ltd., Kyoto, Japan). Creatine was separated by isocratic elution with a mobile phase containing a mixture of 10 mM 1-Octanesulfonic acid (C_8_H_18_O_3_S) water-acetonitrile (95:5 *v*/*v*). The pH of the mobile phase was adjusted to 3.2 with orthophosphoric acid. The flow rate was 1 mL/min, and used by C18 colum and detected at 236 nm [16,17]. Areas under the curve (AUC), Tmax, and Cmax were calculated.

### 2.5. Effect of LMP-Formulation on L-Carnitine and Creatine Bioavailability in Human Subjects

A total of 15 young, healthy male and female subjects were recruited to participate in this study (body mass index (BMI): 18.5–24.9 kg/m^2^) (18–30 years). This study was approved by the Tufts University Health Sciences Investigational Review Board. Study inclusion criteria consisted of normal clinical laboratory values, non-pregnant females, lack of chronic disease and medical clearance by physical examination by the study physician. Participants were recreationally active (≥150 min/week of physical activity) but were not competitive athletes and were instructed not to alter their exercise regimen during the study. Subjects were administered varying doses of LMP Cr (100 mg, 200 mg, and 500 mg) combined with a single dose of LMP CARN (500 mg) in a randomized, double-blind cross-over design, following an overnight 12 h fast. The 200 and 500 mg doses correspond to 21 and 51 mg/kg used in the rodent trial. In addition, capsules of matching weight and appearance consisting of the same LMP formulation but without CARN or Cr were administered to the placebo group. Each subject underwent 4 trials separated by a minimum of a 3-week washout period. Subjects reported to the Metabolic Research Unit in the morning following an overnight (12 h) fast. Subjects’ body weight and vital signs were obtained, and their fasting status was confirmed. A small intravenous catheter was placed in an antecubital vein, and patency was maintained with normal saline. Following baseline blood sampling, subjects consumed one of the following doses at each visit (CARN 500 mg/Cr 100 mg (Dose A), CARN 500 mg/Cr 200 mg (Dose B), CARN 500 mg/Cr 500 mg (Dose C), non-nutritive placebo (Dose D). During each trial, blood samples were obtained at 30, 60, 80, 120, 150, and 300 min. Serum samples were stored at −70 °C for subsequent CARN and Cr analysis.

### 2.6. Serum L-Carnitine and Creatine Analysis

The serum concentrations of L-carnitine and creatine were measured by HPLC-MS/MS (Waters Atlantis HILIC Silica column, 3 µm; 2.1 × 100 mm, with Atlantis HILIC 3 µm 2.1 × 5 mm VanGuard Cartridge, detection via positive electrospray ionization and multiple reaction monitoring) [18]. The analytical method involved the use of a surrogate matrix solution (sodium chloride, potassium chloride, dibasic sodium phosphate, monobasic sodium phosphate, and bovine serum albumin) to help reduce any matrix interference from the plasma samples. External calibration (30 to 1100 ng/mL L-carnitine and creatine) in conjunction with labeled internal standard addition (L-carnitine-(trimethyl-d9)-inner salt and creatine-(methyl-d3) monohydrate) was employed. Matrix blanks, as well as quality control samples, were analyzed with each batch of plasma samples to ensure the proper operation of the method.

### 2.7. Statistical Analysis

For the cell-based experiments, all statistical calculations were performed using unpaired t-tests for protein synthesis and signaling, comparing each group to the control group. Significance was set at *p* < 0.05. Since we were comparing these doses in a hypothesis-generating exploratory fashion, we did not adjust our *p* values for multiple comparisons. For the animal study, data are expressed as the mean ± standard deviation (SD). All statistical calculations were performed using SPSS Statistics 21.0 software (IBM SPSS Inc., Chicago, IL, USA). Statistical significance of differences among groups was evaluated using the One-Way ANOVA test followed by Tukey post hoc test. A value of *p* < 0.05 was considered statistically significant. For the human pharmacokinetic studies, data were expressed as the mean ± standard deviation (SD). Repeated measures analysis was used to test for an interaction between dose and time. Separately for creatine and L-carnitine, repeated measure analysis at the time point with the peak change was used to determine the average difference between placebo and each of the three treatment groups. Similar to the other experiments, *p*-values were not adjusted for multiple comparisons. All analyses were performed using SAS 9.4, and *p* < 0.05 was considered statistically significant.

## 3. Results and Discussion

### 3.1. Effects of L-Carnitine and Creatine on Muscle Protein Synthesis and Anabolic Signaling in Human Primary Myoblasts

**Figure 1:**
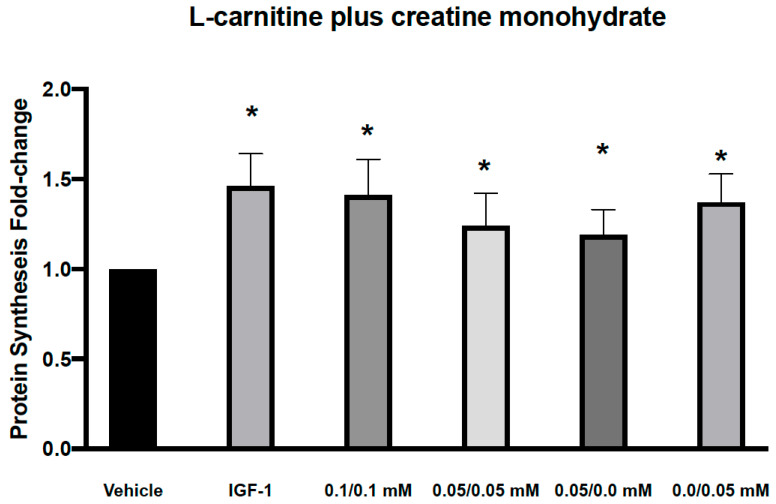
Effects of L-carnitine and creatine at 0.05 and 0.1 mM individually or in combination on muscle protein synthesis. IGF-1 used as a positive control increased protein synthesis by 1.34 ± 0.08 and L-carnitine/Creatine at 0.1/0.1, 0.05/0.05, 0.05/0 and 0/0.05 mM by 1.34 ± 0.09, 1.38 ± 0.04, 1.27 ± 0.1 and 1.13 ± 0.09, respectively from vehicle control. Significance was determined by unpaired *t*-tests. * *p* < 0.05.

**Figure 2:**
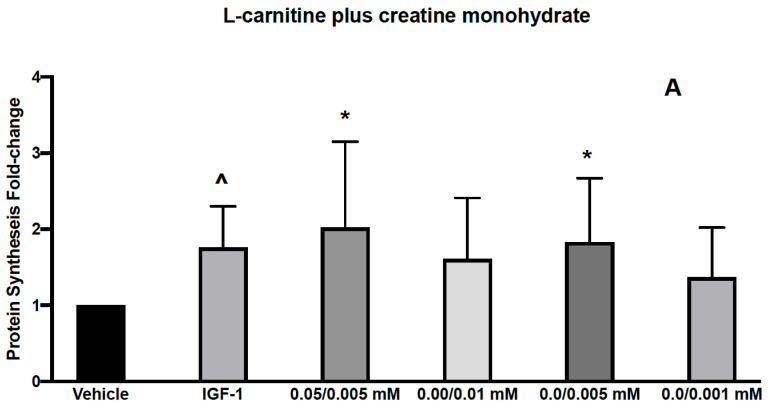

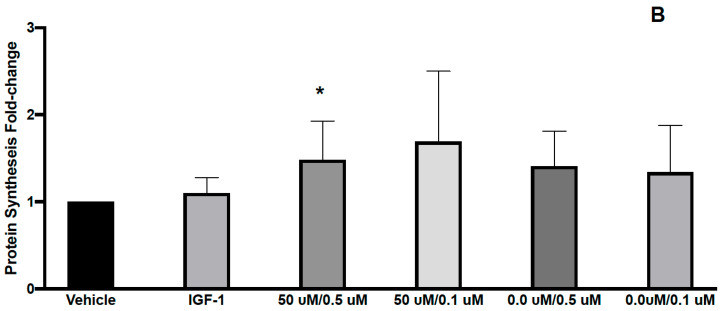
Effects of L-carnitine and creatine at 0.05/0.005 mM and creatine at 0.01, 0.005 and 0.001 mM on muscle protein synthesis (**A**). IGF-1 used as a positive control increased protein synthesis by 1.76 ± 0.22 and L-carnitine/Creatine at 0.05/0.005, 0/0.01, 0/0.005 and 0/0.001 mM by 2.02 ± 0.46, 1.61 ± 0.33, 1.83 ± 0.34 and 1.37 ± 0.27, respectively from vehicle control. Effects of L-carnitine and creatine at 50 μM/0.5 μM and 50 μM/0.1 μM and creatine at 0.5, 0.1 μM were assessed for their effects on muscle protein synthesis (**B**). IGF-1 used as a positive control increased protein synthesis by 1.1 ± 0.07 and L-carnitine/Creatine at 50/0.5, 50/0.1, 0/0.5, 0/0.1 mM by 1.48 ± 0.17, 1.69 ± 0.29, 1.41 ± 0.15 and 1.34 ± 0.2, respectively from vehicle control. Significance was determined by unpaired *t*-test. * *p* < 0.05, ^ *p* < 0.01.

We first assessed the effect of 2 combinations of CARN/Cr at 0.05 mM/0.05 mM or 0.1 mM/0.1 mM on muscle protein synthesis in human primary myoblasts. Myoblasts, although not fully differentiated, are growth-factor responsive and have been used in similar experiments previously [19,20,21].These doses were chosen based on the reported plasma levels for these compounds. We found that both combinations led to a significant increase in protein synthesis (Figure 1). However, individually, both L-carnitine and creatine at 0.05 mM also led to similar significant increases in protein synthesis, suggesting that these are not the minimal doses required to activate muscle protein synthesis (Figure 1). We next assessed the effects of lower doses, 0.05 mM L-carnitine with 0.005 mM creatine in combination (Figure 2A). We found that this combination led to a significant increase in protein synthesis, as did Cr alone at 0.005 mM (Figure 2A). Interestingly, the higher dose of creatine alone (0.01 mM) did not increase protein synthesis. It has been reported that creatine-induced protein synthesis in the C2C12 muscle cell line follows a bell-shaped curve and drops with high concentrations of creatine [22]. This phenomenon was explained by the inability of the cells to regulate their volume at higher concentrations of creatine and make more protein during their growth. The increase in protein synthesis at CARN/Cr of 0.05/0.005 mM was also translated to a significant rise in p-AKT but not p-RPS6 phosphorylation (Table 1). It has been reported that creatine at 5 mM induced myoblast differentiation by an increase in p38 and p-AKT in the C2 C12 cell line [22]. When Cr at 0.5 μM was used in combination with CARN 50 μM, a significant effect on protein synthesis was seen (Figure 2B), without significant effects on the signaling pathway (Table 1). Cr at 0.5 μM did result in a significant change in protein synthesis. Interpreted together, protein synthesis and signaling data suggest a synergistic response of CARN and Cr at 50 μM and 0.5 μM, respectively (Figure 2B). Effects on anabolic signaling, however, were observed only at CARN 0.05 mM and CRE 0.005 mM in combination (Table 1).

### 3.2. Effect of Lipid Multi-Particulate Formulated Creatine on Serum Bioavailability in Rodents

**Figure 3:**
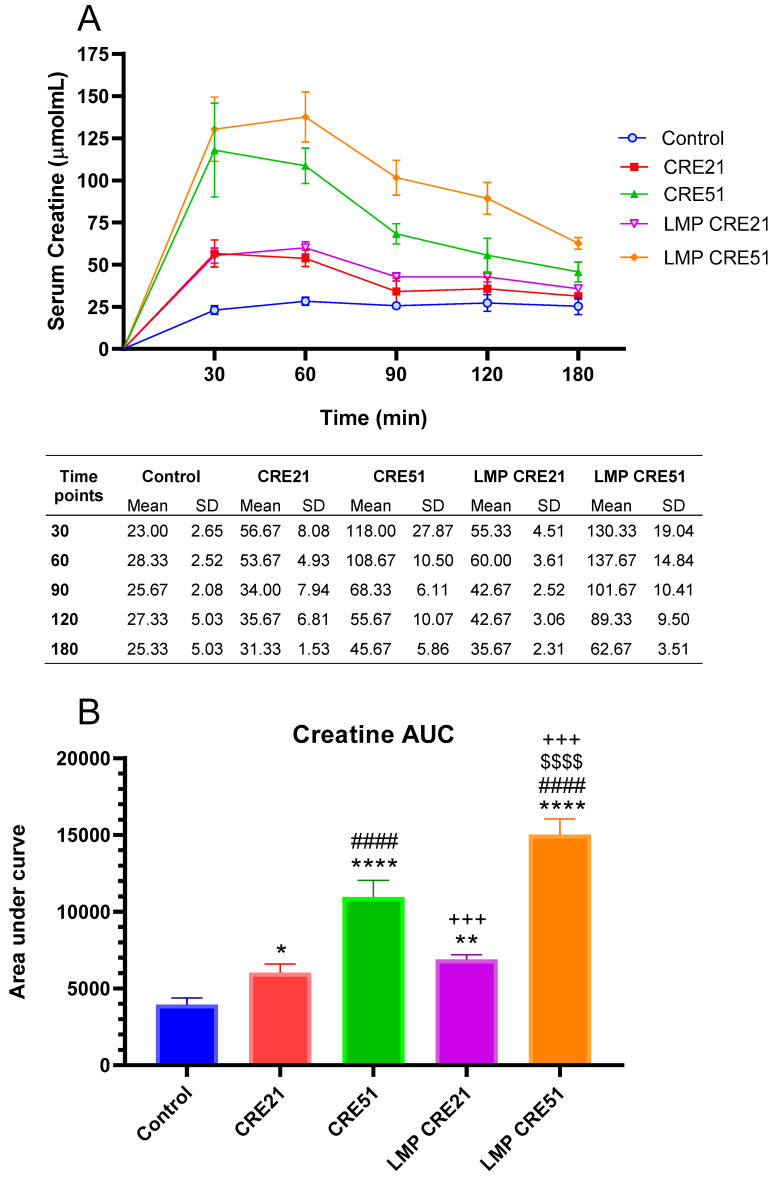
Effect of LMP formulation on serum creatine concentration in rodents. Serum creatine concentration following oral administration at 21 mg/kg (CRE21) or 51 mg/kg (CRE51) (*n* = 7) without or with LMP (LMP CRE21 and LMP CRE51, respectively) in rat sera (**A**). Area Under Curve (AUC) for creatine with or without LMP in rat sera (**B**). The error bars correspond to the standard deviation of the mean. In Panel B, ANOVA and the Tukey post-hoc test were used for comparing the results among different treatment groups. Statistical significance between groups is shown by * *p* < 0.05; ** *p* < 0.01; **** *p* < 0.0001 as compared to control group, #### *p* < 0.0001 as compared to CRE 21 group, +++ *p* < 0.001 as compared to CRE 51 group, and $$$$ *p* < 0.0001 as compared to LMP CRE 21 group.

The analytical peaks of creatine with or without LMP formulation along with internal standard were resolved with good symmetry in blank plasma. Creatine administered alone or in LMP formulation was detected in all serum samples. Mean serum concentration-time profiles of creatine with or without LMP formulation are shown in Figure 3A. Serum creatine concentrations from each formulation at 21 mg/kg creatine (CRE21) and 51 mg/kg creatine (CRE51) doses achieved the greatest mean levels at 30 min without LMP formulation and 60 min when formulated in LMP (LMP CRE21 and LMP CRE51). These data suggest that LMP formulation increased creatine absorption, most likely by diffusion through the intestinal wall. Serum creatine concentrations were the greatest in rats orally administered a higher dose of creatine LMP (LMP CRE51) at all times measured. The high creatine dose formulated in LMP (LMP CRE51) achieved the highest availability and reached its maximum concentration levels at 60 min and did not decrease until 90 min. LMP CRE51 was significantly more available than CRE51. As expected, the high creatine dose (51 mg/kg) with or without LMP provided higher serum creatine concentrations than the lower dose (21 mg/kg). Similar results were also observed with the AUC data (Figure 3B), where the highest availability was obtained with the high creatine dose in LMP, LMP CRE51, which had 27% higher bioavailability than CRE21 (*p* = 0.0004). The formulation with LMP at the lower dose of creatine (21 mg/kg), LMP CRE21, did not change the AUC significantly compared to the same dose without LMP (*p* *>* 0.05). These data suggest that LMP formulation increases the bioavailability of creatine when given at 51 mg/kg. Time to peak plasma concentration (Tmax) for oral creatine and maximum concentration (Cmax) were not significantly affected by LMP formulation for either dose (data not shown). The AUC reflects the total plasma exposure of creatine and is a measure of its bioavailability. Tmax and Cmax, however, represent the rate of absorption. Therefore, it can be concluded that LMP formulation does not affect the rate of absorption but increases plasma exposure of creatine at 51 mg/kg. The effects of LMP formulation on creatine bioavailability were seen at a dose of 51 mg/kg, corresponding to 500 mg daily in humans. Creatine is absorbed actively in the gut. Its clearance can increase due to the saturation of its transport system, particularly at high doses or with chronic supplementation because of the reported skeletal muscle store saturation [23]. Therefore, technologies such as a lipid multi-particulate can facilitate its slow diffusion when the active transporter is saturated. This observation supports the suggestion that LMP formulation increased creatine bioavailability only at a high dose.

### 3.3. Effect of Lipid Multi-Particulate Formulated L-Carnitine and Creatine on Serum Bioavailability in Humans

To assess the optimal doses of the LMP CARN and LMP Cr combination on bioavailability in humans, a total of 15 healthy subjects were enrolled in a pilot randomized double-blinded placebo-controlled cross-over design trial. The population included 68% females with an average age of 21.9 years and a BMI of 22.0 kg/m^2^. Nine subjects completed the four trials. Six subjects withdrew from the study before completing all four trials due to scheduling constraints or loss of interest. No adverse events were reported.

**Figure 4:**
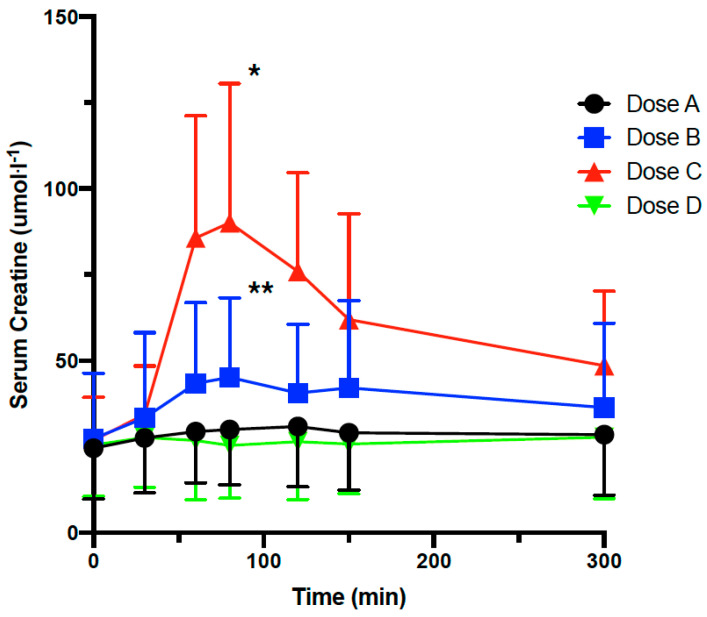
Pharmacokinetic of LMP formulated creatine in humans in a randomized double-blinded placebo-controlled cross-over study. Serum creatine concentration following oral administration of placebo or LMP CARN (500 mg) combined with LMP Cr at 100, 200 or 500 mg in 17 healthy individuals. Dose A = LMP CARN 500 mg/LMP Cr 100 mg, Dose B = LMP CARN 500 mg/LMP Cr 200 mg, Dose C = LMP CARN 500 mg/LMP Cr 500 mg, Dose D = non-nutritive placebo. Data are shown as mean and standard deviation (above or below). ** indicates Dose B significantly different from Dose D at 90 min (*p* = 0.01). * indicates Dose C is significantly different from Dose D at 90 min (*p* < 0.0001).

Participants were randomized to 4 treatment groups as following: Dose A (LMP CARN 500 mg/LMP Cr 100 mg), Dose B (LMP CARN 500 mg/LMP Cr 200 mg), Dose C (LMP CARN 500 mg/LMP Cr 500 mg) and Dose D (non-nutritive placebo). Serum L-carnitine and creatine levels were measured. Serum creatine levels rose significantly in response to Doses B and C, with the greatest increase observed with Dose C at 80 min following dosing based on the changes in the AUC (Dose A was 4.7 μmol·L^−1^ higher than Dose D (*p* = 0.51); Dose B was 19.0 μmol·L^−1^ higher than Dose D (*p* = 0.01); Dose C was 61.1 μmol·L^−1^ higher than Dose D (*p* < 0.0001)) (Figure 4). Overall, serum total L-carnitine levels rose in a similar manner following Dose A, B, and C, with peak levels occurring at 300 min (data not shown). Dose A was 1.8 μmol·L^−1^ higher than Dose D (*p* = 0.35); Dose B was 6.3 μmol·L^−1^ higher than Dose D (*p* = 0.004); Dose C was 5.2 μmol·L^−1^ higher than Dose D (*p* = 0.02)). Creatine is actively transported, and its absorption, distribution and clearance depend on the doses supplemented and the integrity of its transporter (SLC6A8) [23]. It has been reported that deficiency in SLC6A8 levels leads to lower creatine availability in the muscle [24]. In our study, acute administration of 200 or 500 mg of creatine led to a maximum serum concentration of about 45 and 90 μmol·L^−1^, respectively. Assuming that LMP-formulated creatine absorption and its appearance in the blood are linear, acute administration of 5 g would result in a serum concentration of 900 μmol·L^−1^. However, this assumption needs to be confirmed in a clinical trial using the LMP-formulated creatine. Previously, it was reported that acute administration of 5 g creatine resulted in a maximum concentration of 511 μmol·L^−1^ among young subjects and 664 μmol·L^−1^ among older men [25]. Taken together, LMP formulation seems to increase bioavailability in humans by 26 to 40%, a level comparable to what we found in rodents (27% increase). These data suggest that LMP formulation increases in serum bioavailability of creatine is species-independent.

## 4. Conclusions

This study indicates that a combination of CARN and Cr at doses of 50 μM and 0.5 μM, respectively, increased protein synthesis in human primary myoblasts, but the effects on anabolic signaling were observed only at 50 μM and 5 μM doses, respectively. The same doses, individually, did not lead to any of these effects, suggesting that the effects of CARN and Cr are synergistic. In addition, based on the rodent bioavailability and human pharmacokinetic trials, LMP formulation seems to enhance creatine bioavailability at 500 mg dose/day human equivalent. L-carnitine, however, did not benefit from the LMP formulation. These data suggest that L-carnitine at 500 mg and creatine at 200 or 500 mg formulated in LMP may increase bioavailability in human subjects. This has the potential to synergistically enhance muscle protein synthesis and anabolic signaling based on in vitro evidence. Further clinical trials are warranted to examine these questions.

## Figures and Tables

**Table 1:** Phospho-AKT and RPS6 expression in response to varying doses of L-carnitine (CARN) and creatine (Cr). IGF-1 is positive control.

	IGF-1		0.05/0.005 mM	0.0/0.01 mM	0.0/0.005 mM	0.0/0.001 mM
p-AKT	1.65 (0.50) *p* < 0.05	CARN/Cr	1.46 (0.40) *p* < 0.05	1.13 (0.31)	1.26 (0.31)	1.06 (0.28)
p-RPS6	1.39 (0.41) *p* < 0.05		1.41 (0.53) *p* < 0.10	1.07 (0.32)	1.31 (0.44)	1.14 (0.46)
		CARN/Cr	50 μM/0.5 μM	50 μM/0.1 μM	0.0 μM/0.5 μM	0.0 μM/0.1 μM
p-AKT	1.37 (0.28) *p* < 0.05		1.08 (0.41)	1.16 (0.40)	1.20 (0.47)	1.28 (0.31)
p-RPS6			0.84 (0.27)	0.87 (0.25)	0.87 (0.22)	0.93 (0.16)

## Data Availability

The data presented in this study are available on request from the corresponding author. The data are not publicly available due to privacy and ethical issues.
